# The future of mechanical ventilation: lessons from the present and the past

**DOI:** 10.1186/s13054-017-1750-x

**Published:** 2017-07-12

**Authors:** Luciano Gattinoni, John J. Marini, Francesca Collino, Giorgia Maiolo, Francesca Rapetti, Tommaso Tonetti, Francesco Vasques, Michael Quintel

**Affiliations:** 1 Department of Anesthesiology, Emergency and Intensive Care Medicine, University of Göttingen, Robert-Koch-Straße 40, 37075 Göttingen, Germany; 2 University of Minnesota, Minneapolis/Saint Paul, MN USA

**Keywords:** Mechanical ventilation, Acute respiratory distress syndrome, Ventilator-induced lung injury, Mechanical power, Extracorporeal membrane oxygenation

## Abstract

The adverse effects of mechanical ventilation in acute respiratory distress syndrome (ARDS) arise from two main causes: unphysiological increases of transpulmonary pressure and unphysiological increases/decreases of pleural pressure during positive or negative pressure ventilation. The transpulmonary pressure-related side effects primarily account for ventilator-induced lung injury (VILI) while the pleural pressure-related side effects primarily account for hemodynamic alterations. The changes of transpulmonary pressure and pleural pressure resulting from a given applied driving pressure depend on the relative elastances of the lung and chest wall. The term ‘volutrauma’ should refer to excessive strain, while ‘barotrauma’ should refer to excessive stress. Strains exceeding 1.5, corresponding to a stress above ~20 cmH_2_O in humans, are severely damaging in experimental animals. Apart from high tidal volumes and high transpulmonary pressures, the respiratory rate and inspiratory flow may also play roles in the genesis of VILI. We do not know which fraction of mortality is attributable to VILI with ventilation comparable to that reported in recent clinical practice surveys (tidal volume ~7.5 ml/kg, positive end-expiratory pressure (PEEP) ~8 cmH_2_O, rate ~20 bpm, associated mortality ~35%). Therefore, a more complete and individually personalized understanding of ARDS lung mechanics and its interaction with the ventilator is needed to improve future care. Knowledge of functional lung size would allow the quantitative estimation of strain. The determination of lung inhomogeneity/stress raisers would help assess local stresses; the measurement of lung recruitability would guide PEEP selection to optimize lung size and homogeneity. Finding a safety threshold for mechanical power, normalized to functional lung volume and tissue heterogeneity, may help precisely define the safety limits of ventilating the individual in question. When a mechanical ventilation set cannot be found to avoid an excessive risk of VILI, alternative methods (such as the artificial lung) should be considered.

## Background

For a reasonable number of years to come, mechanical ventilation will likely still be needed. We acknowledge the importance of stabilizing hemodynamics [1], achieving synchrony [2], preserving muscle strength [3, 4], avoiding the consequences of intubation [5], minimizing dynamic hyperinflation [6], and monitoring the biological reactions—all important goals of ventilatory support. In this brief review, however, we focus primarily on limiting tissue damage, thereby improving the safety of artificial ventilation. Further we will limit our analysis to ARDS patients, who are among the most problematic to manage among the mechanically ventilated patients. However, the principles of a safe treatment are equally applicable to all mechanically ventilated patients. To artificially inflate the lung (i.e., to increase the transpulmonary pressure (*P*_L_), airway pressure – pleural pressure (*P*_aw_ – *P*_pl_)), two diametrically opposed options can be applied: either totally positive airway pressure ventilation associated with an increase of pleural pressure or totally negative pressure ventilation, in which the chest cage is expanded by external negative pressure. Between these two extremes, mixed forms of ventilation may be applied, primarily by providing positive pressure to the airways while allowing spontaneous contraction of the respiratory muscles, which decrease pleural pressure during inspiration (Table 1). To discuss the future we must first understand the current problems associated with mechanical ventilation.

**Table 1:** ‘Motors’ of the lung and chest wall during positive and negative ventilation

	Positive pressure ventilation	Negative pressure ventilation	
		Spontaneous	Artificial
Respiratory system motor	Energy from ventilator generating the airway pressure (*P*_aw_)	Energy from muscular contraction generating muscular pressure (*P*_musc_)	Energy from device generating negative pressure (*P*_neg_)
Lung motor	Transpulmonary pressure (*P*_L_) generated by positive increase of *P*_aw_ and pleural pressure (*P*_pl_)	Transpulmonary pressure (*P*_L_) generated by decrease of pleural pressure (*P*_pl_)	Transpulmonary pressure (*P*_L_) generated by decrease of pleural pressure (*P*_pl_)
Chest wall motor	Pleural pressure (*P*_pl_ = *P*_aw_ – *P*_L_)	Wall pressure^a^ (*P*_W_ = *P*_musc_ – *P*_pl_)	Wall pressure^a^ (*P*_W_ = *P*_neg_ – *P*_pl_)

^a^Wall pressure is the component of total muscular (or externally applied negative pressure) needed to expand the chest wall itself

## Adverse effects of mechanical ventilation

**Fig. 1:**
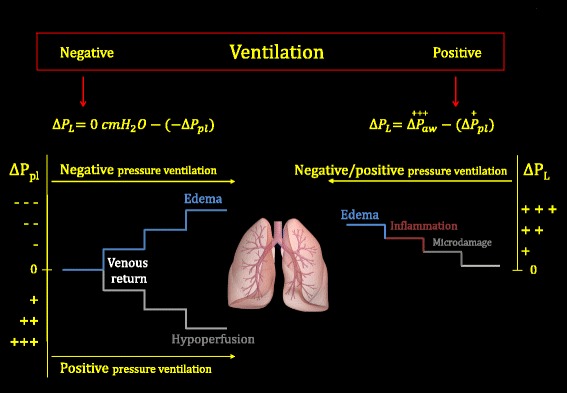
Changes of transpulmonary pressure (∆*P*_L_) and of pleural pressure (∆*P*_pl_) during negative or positive pressure ventilation. *Left*: possible adverse consequences due to the progressive decrease or progressive increase of pleural pressure (∆*P*_pl_). The key variation is the increase or decrease of venous return, respectively. *Right*: sequence of possible damage when progressively increasing the transpulmonary pressure (∆*P*_L_). Either during negative pressure ventilation (here performed at baseline atmospheric pressure, i.e., 0 cmH_2_O) or during positive pressure ventilation, ∆*P*_L_ is always positive. See text for details. ∆*P*_aw_ change in airway pressure

The adverse effects of mechanical ventilation may be grouped into two main categories. One category relates to excessive/unphysiological transpulmonary pressure (always positive), and the other relates to excessive/unphysiological variation of pleural pressure, either positive or negative (Fig. 1).

### Side effects associated with pleural pressure

The magnitude and direction of change in pleural pressure, negative or positive, depends on the ratio of chest wall elastance (*E*_W_) relative to the elastance of the respiratory system (*E*_tot_). The latter equals the sum of the chest wall elastance and the lung elastance (*E*_L_). Accordingly, during positive pressure ventilation the following relationship applies under static conditions [7]:

1 $$ \varDelta {P}_{\mathrm{pl}}=\varDelta {P}_{\mathrm{aw}}\cdot \frac{E_{\mathrm{w}}}{E_{\mathrm{tot}}} $$

During negative pressure ventilation, however, where the inflation-producing change in pressure is a reduction in the pressure surrounding the respiratory system (Δ*P*neg), the following applies:

2 $$ -\varDelta {P}_{\mathrm{pl}}=\varDelta {P}_{\mathrm{neg}}\cdot \frac{E_{\mathrm{w}}}{E_{\mathrm{tot}}} $$

Note that, in ARDS, the *E*_W_/*E*_tot_ ratio averages 0.7, but may range from 0.2 to 0.8 [8].

Obviously, in the presence of an artificial ventilation mode where positive pressure may work simultaneously with muscular efforts ($\Delta {P}_{musc}\Big)$ (Table 1), the actual changes of pleural pressure result from two ‘push–pull’ forces. Accordingly:

3 $$ \varDelta {P}_{pl}=\varDelta {P}_{\mathrm{aw}}\cdot \frac{E_{\mathrm{w}}}{E_{\mathrm{tot}}}-\varDelta {P}_{\mathrm{musc}}\cdot \frac{E_{\mathrm{L}}}{E_{\mathrm{tot}}} $$

#### Positive pleural pressure

For passive inflation by a given airway pressure, the pleural pressure will increase far more in the presence of elevated chest wall elastance (i.e., elevated *E*_W_/*E*_tot_), as in some cases of extreme obesity [9], whereas it will increase far less in the presence of elevated lung elastance (i.e., low *E*_W_/*E*_tot_; see Eq. (1)). All equations to which we refer only approximate what is actually happening in the pleural space, because in reality the pleural pressure is not uniform along the thoracic cage, but rather depends on several factors, such as gravitational gradients and local pressure distortions arising from anatomical differences in the shapes of the lung and its chest wall enclosure [10]. Despite the limitations in accurately determining pleural pressure [11, 12], its changing value influences central vascular pressures and venous return. A large experimental and clinical literature describes all of the possible complications related to ventilation-caused decreases of effective circulating volume. These are particularly likely to occur when pleural pressure remains positive throughout the entire respiratory cycle, as during ventilation with positive end-expiratory pressure (PEEP) [13]. The kidney [14], liver [15], and bowel [16, 17] may all be impaired or damaged by the resulting venous congestion and reduced perfusion.

#### Negative pleural pressure

Excessively negative pleural pressure may arise during spontaneous breathing, especially when vigorous respiratory effort is applied to a ‘stiff lung’ (see Eq. (3)). In ARDS, for example, negative swings in esophageal pressure may exceed 20–25 cmH_2_O, due to profoundly dysregulated respiratory drive [18]. Apart from increasing the work of breathing and oxygen consumption, such excessively negative intrathoracic and interstitial pressures promote venous return and increase edema formation. Such phenomena, well described by Barach et al. in 1938 [19], have deservedly been reemphasized for the current era of positive pressure ventilation [20]. Recent work has demonstrated that pedelluft phenomena which occur during vigorous breathing efforts in injured lungs have the potential to amplify local strains and could conceivably contribute to tissue damage [21–23]. In concept, certain asynchronies between the patient and ventilator (e.g., double triggering and breath stacking) may also be injurious when they occur frequently and/or in groups.

### Adverse effects associated with transpulmonary pressure

The adverse effects of excessive transpulmonary pressure were recognized soon after mechanical ventilation was first applied in patients with ARDS [24]. In those early years the initial therapeutic targets were to maintain normal blood gases and to avoid dyssynchrony while limiting the use of muscle relaxants, which understandably were considered hazardous when using the poorly alarmed ventilators of that era. Consequently, tidal volumes and respiratory rates were typically 15 ml/kg and 15–20 bpm, respectively [25]. Using this approach, few patients fought the ventilator, but barotrauma (primarily pneumothorax) occurred quickly and commonly. This event was so frequent that preventive use of bilateral chest tubes was suggested when ventilation for ARDS was initiated [26]. ‘Barotrauma’ was used to collectively identify the clinically recognizable problems of gas escape: pneumothorax, pneumomediastinum, interstitial emphysema [27–30], gas embolism [31], etc. Used in a broader sense, however, barotrauma also includes VILI.

A different viewpoint was elaborated by Dreyfuss et al. [32], who emphasized the role of lung distention (strain) as opposed to airway pressure. High airway pressures were applied without excessive lung strain or damage by restricting chest wall movement. Conversely, injury (‘volutrauma’) was inflicted by similar airway pressures in the absence of chest wall restraint. Barotrauma and volutrauma, however, are two faces of the same coin if we consider that the force distending the lung is not the airway pressure, but the transpulmonary pressure (i.e., *P*_aw_ – *P*_pl_). This variable more accurately reflects the stress applied to lung structures. Indeed, the following relationship holds [7]:

4 $$ {P}_{\mathrm{L}}={E}_{Lspec}\cdot \frac{\varDelta V}{FRC} $$

Here, $\Delta V$ is the change in lung volume in reference to its resting (unstressed) value, functional residual capacity (FRC), and ${E}_{Lspec}$ is the tissue elastance of the lung, elastance referenced to the lung’s absolute inflation capacity.

In words, Eq. (4) can be expressed as:

5 $$ S t r e s s={E}_{Lspec}\cdot S t r a i n $$

implying:

6 $$ B a r o t r a u m a= k\cdot V o l u t r a u m a $$

**Fig. 2:**
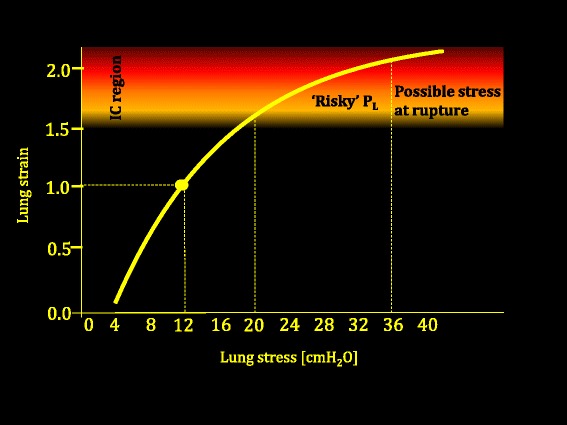
Lung strain (tidal volume/FRC) as a function of lung stress (transpulmonary pressure). Data adapted from Agostoni and Hyatt [74]. As shown, the doubling of the FRC occurs at a transpulmonary pressure of 12 cmH_2_O (specific elastance). We arbitrarily indicated the ‘risky’ zone of *P*_L_ as that which corresponds to lung strains exceeding 1.5 (based on experimental data [52]). *P*_L_ transpulmonary pressure

Therefore, stress and strain are related by a proportionality constant, equivalent to specific elastance ${E}_{Lspec}$. This value, which is similar in normal subjects and in acute lung injury patients, averages ~12 cmH_2_O [8]. In other words, 12 cmH_2_O is the stress developed in lung structures when the resting volume (FRC) is doubled. Indeed, at total inspiratory capacity the stress would be ~24 cmH_2_O because the ∆V/FRC ratio is then ~2. Experimental studies indicate that barotrauma/volutrauma requires some regions of the lung to reach the ‘their own’ total lung capacity [33]. At this level, the collagen framework is fully distended and works as a ‘stop length’ restraint. These concepts are summarized in Fig. 2 and form a basis for understanding barotrauma and volutrauma.

#### Volutrauma

In comparative studies investigating the role of volutrauma on outcome, tidal volume has usually been expressed per kilogram of ideal (predicted) body weight (PBW) in an attempt to relate tidal volume to the expected lung size. Unfortunately, due to the variability of the aeratable lung size in ARDS (the concept of ‘baby lung’ [34]), such normalization fails as a surrogate for lung strain. Despite these limitations, the ARDS Network [35] found a 9% survival benefit in an unselected ARDS sample when using 6 ml/kg PBW tidal volume instead of 12 ml/kg PBW. Of note, this advantage was also found in the quartile of patients with less severe ARDS, where the ‘baby lung’ size was likely greater [36]. It seems plausible that the inverse correlation between survival and dead space [37], as reflected by hypercapnia, may relate to the relative sizes of the functioning baby lungs and the strains that they undergo with ‘lung protective’ ventilation [38]. A tidal volume per kilogram exceeding 20–30 ml/kg is required to damage the healthy lungs of experimental animals [39–43]. Although a direct comparison between healthy and ARDS lungs is highly questionable, the mechanical characteristics of the ‘baby lung’ (i.e., its specific compliance) are similar to those of normal subjects. The ARDS Network mandate to avoid high tidal volumes deeply and appropriately influenced clinical practice. However, volutrauma may best be avoided by considering not simply the tidal volume but the strain (i.e., the ratio of tidal volume to the resting lung volume). In this context, the recently redirected focus on driving pressure (which equals the ratio of tidal volume to compliance) rather than on plateau pressure alone has a rough parallel with this admonition [44]. We must also remind ourselves that in prior randomized controlled trials [45–47], the ARDS patients exposed to ~10 ml/kg tidal volume experienced better survival compared to patients exposed to ~7 ml/kg. Therefore, decreases of tidal volume below 6 ml/kg, as proposed for ‘ultraprotective ventilation’ (associated with extracorporeal CO_2_ removal) would not necessarily be of benefit, because severe hypoventilation and reabsorption atelectasis may offset its putative advantages unless other preventative or compensatory measures are taken to raise mean airway pressure, with consequent increase of global lung stress [48, 49]. Attention should be paid to avoiding not only excessively high strain, but also unphysiologically low strain.

#### Barotrauma

In the editorial accompanying the ARMA trial, 32 cmH_2_O plateau pressure was suggested as an upper safety limit for (passive) mechanical ventilation [50]. Since then, the 30 cmH_2_O limit became infrequently challenged dogma for both clinical practice and clinical trials. Actually, in a normal 70-kg human (FRC ~2000 ml and compliance ~80 ml/cmH_2_O), the 30 cmH_2_O plateau would correspond to a tidal volume of ~2400 ml (strain = 1.2). In normal animals, this strain is nearly harmless if applied at a respiratory rate of 15 bpm for 54 hours [51]. The applied transpulmonary pressure in this condition, assuming similar chest wall and lung elastances, would be ~15 cmH_2_O (see Fig. 2). However, as already stated, in ARDS the ratio between lung elastance and the total respiratory system elastance may vary from 0.2 to 0.8 [8]. Because the transpulmonary pressure equals the applied airway pressure times the *E*_L_/*E*_tot_ ratio, the ‘safe’ 30 cmH_2_O may result in a transpulmonary pressure as low as 6 cmH_2_O or as high as 24 cmH_2_O, a value approaching that needed to reach total lung capacity (Fig. 2), and may be lethal to animals [52]. Therefore, the use of 30 cmH_2_O, in a given subset of patients may result either in excessive strain or in hypoventilation and hypoxemia. This was likely the case of many patients with low *E*_L_/*E*_tot_ ratios (i.e., pregnant women or obese patients) during the H1N1 epidemics in Australia and New Zealand [53]. In some of those patients, ECMO perhaps could have been avoided, simply by safely increasing the plateau pressure, as we found in a cohort of H1N1 patients (ECMO candidates), where low *E*_L_/*E*_tot_ was documented [54]. Just as for volutrauma it is wiser to consider strain instead of the tidal volume, for barotrauma it is wiser to consider transpulmonary pressure instead of plateau airway pressure (see Eq. (6)).

### Consequences associated with other ventilatory variables

Although most of the studies dealing with VILI concentrate on the static components of the breath (tidal volume, plateau pressure, and PEEP), other important factors should not be ignored. The most relevant, in our opinion, are the respiratory rate (i.e., how many times per minute a potential volutrauma or barotrauma is delivered) and the inspiratory flow rate (i.e., how fast a potential volutrauma or barotrauma is applied).

#### Respiratory rate

The respiratory rate has been considered relatively inconsequential, because it is usually set to maintain PaCO_2_ within an acceptable range. Thus, in the milestone ARDS Network trial, the lower tidal volume was associated with a respiratory rate of 29 bpm, compared to 16 bpm in the higher tidal volume group. Nonetheless, under certain conditions the respiratory rate is unlikely to be innocent in the genesis of VILI. The harm resulting from raising the respiratory rate is almost certain to be conditioned by the dynamic stress of the individual tidal cycle [55]. The analogy with metal fatigue, which is a function of the number of high stress cycles, may help to frame the role of respiratory rate as codeterminant of VILI. Both in isolated lungs and large-size animals, reducing the respiratory rate provides definite advantages in reducing VILI [56, 57]. Conversely, when operated in an elevated pressure range, perhaps high-frequency ventilation with small tidal volumes may inflict damage [58].

#### Inspiratory flow

The potential for high inspiratory flow to contribute to VILI likely relates to locally intensified concentration of stress, a problem influenced by viscoelastic tissue properties. Experimental literature consistently shows that, for a given plateau pressure, or a given strain, the rate at which the volume was delivered (i.e., the inspiratory flow) plays a definite role in the genesis of VILI [33, 59–61]. Although one would logically expect that any damage attributed to high inspiratory flow should primarily concentrate in the airway, high inspiratory flow accentuates damage to the lung parenchyma, in all likelihood because viscoelastic accommodation has insufficient time to dissipate damaging forces when inflation occurs quickly. Flow rate assumes a greater role in a mechanically inhomogeneous lung (e.g., ARDS) than in a homogeneous one. Moreover, a tidal volume delivered by pressure control could be more dangerous than if achieved by flow-controlled, volume-cycled ventilation with constant flow, because in the former the peak inspiratory flow may reach far higher values. Finally, although little studied, control of expiratory flow may potentially attenuate microatelectasis and influence stresses that occur as tissues rearrange themselves during deflation.

## Present-day mechanical ventilation

Table 2 presents ventilatory data and outcomes of different populations treated over the years for ARDS. The observational studies presented are the 2002 study by Esteban et al. [62], the 2011 study by Villar et al. [63], and the 2016 study by Bellani et al. [64]. These three studies include unselected ARDS patients and should reflect daily practice. For comparison, we added the ventilatory treatments and outcomes of patients enrolled in randomized trials, filtered through exclusion criteria from a wider ARDS population. In comparison to tidal volume, more attention seems to have been paid to the plateau pressure, which has been held consistently below 30 cmH_2_O after the ARDS Network ARMA trial. The respiratory rate did not change remarkably, because it seems to be dictated by the aim of maintaining PaCO_2_ within normal limits of 35–45 mmHg. PEEP values consistently averaged 7–8 cmH_2_O, with levels up to 15 cmH_2_O systematically applied only in clinical trials. Considering the ventilatory data reported in the largest and most recent survey by Bellani et al. [64], we may wonder what mortality fraction is attributable to VILI in patients ventilated with tidal volume of 7.6 ml/kg PBW, respiratory rate of 18.6 bpm, and PEEP of 8.4 cmH_2_O. To date, we do not believe it is possible to answer this question, which is of paramount importance in improving future mechanical ventilation. Indeed, if the mortality attributable to VILI is now already very low, we cannot expect any great improvement from modifying our current ventilatory practice. We must first better understand the roles played by the mechanical ventilator’s settings, the underlying lung pathophysiology, and their interaction.

**Table 2:** Mechanical ventilation settings through the years

	Brochard et al. [45]		Stewart et al. [46]		Brower et al. [47]		ARDS Network [35]		Esteban et al. [62]	Brower et al. [75]		Meade et al. [76]		Briel et al. [77]		Villar et al. [63]	Guerin et al. [73]	Bellani et al. [64]
Type	RCT		RCT		RCT		RCT		Observation	RCT		RCT		Meta-analysis		Observation	RCT	Observation
Year	1998		1998		1999		2000		2002	2004		2008		2010		2011	2013	2016
Number of patients	58	58	60	60	100	100	387	405	231	236	258	508	475	1163	1136	255	229^a^	2377
Vt/PBW (ml/kg)	7.1	10.3	7.0	10.7	10.2	7.3	6.2	11.8	8.7	6.1	6	6.8	6.8	6.3	6.3	7.2	6.1	7.6
RR (bpm)			22.1	15.6			29	16	20	29	29	26	25.2				27	20.8
Peak pressure (cmH_2_O)			24.2	32.1			32	39	34									27
Plateau pressure (cmH_2_O)	25.7	31.7	22.3	26.8	30.6	24.9	25	33	28	24	27	24.9	30.2	23	29	26	23	23
PEEP (cmH_2_O)	10.7	10.7	8.6	7.2	~8.2	~9.5	9.4	8.6	8	8.9	14.7	10.1	15.6	9	15	9.3	10	8.5
Mortality (%)	46.6^b^	37.9^b^	50^c^	47^c^	46^c^	50^c^	31^c^	39.8^c^	52^c^	24.9^c^	27.5^c^	32.3^d^	28.4^d^	36.6^c^	30.3^c^	42.7^c^	32,8^d^	35.3^c^

*PBW* predicted body weight, *PEEP* positive end-expiratory pressure, *RCT* randomized controlled trial, *RR* respiratory rate, *Vt* tidal volume

^a^ Supine group only

^b^ Fourteen-day mortality

^c^ ICU/hospital mortality

^d^ Twenty-eight-day mortality

## The future of mechanical ventilation

Ideally, mechanical ventilation should be applied so as to avoid all adverse side effects, including VILI. To rationally approach this task, we believe it necessary to characterize much better than we do now the pathophysiology of the lung parenchyma to which the mechanical ventilation is applied and to fully understand the potential harm of each component of the ventilatory set.

### Lung-related causes of VILI

The primary conditions influencing the occurrence of VILI are baby lung size, parenchymal recruitability, and extent of lung inhomogeneity. The routine measurement of the lung size would allow the assessment of average lung strain. The precise assessment of recruitability, which currently requires imaging techniques, will facilitate both increasing functional lung size and preventing/limiting atelectrauma by selecting ‘adequate’ PEEP. Lung inhomogeneity likely promotes VILI. In healthy animals, VILI requires tidal volumes as high as 30–40 ml/kg [39–43, 51]. In contrast, 12 ml/kg appear sufficient, in ARDS patients, even in those with better lung compliance (i.e., with likely greater lung size) [36]. Because the possible alterations within the baby lung (i.e., a deficit of surfactant, the presence of some edema, and fibrosis in the extracellular matrix) are per se protective against excessive strain, additional factors seem necessary to account for the damage. These may be the lung parenchyma inhomogeneities that locally increase the stress and strain (stress raisers). In the classic theoretical model of Mead et al. [65], the inhomogeneity occurring at the interface between a fully open unit (volume = 10) and a fully closed unit (volume = 1) will cause a pressure rise proportional to the exponent 2/3 of their ratio (i.e., (10/1)^2/3^). The proposed exponent of 2/3 is an approximation to convert volume (cm^3^) to surface area (cm^2^), as stress relates to surface area (force divided by surface area). Because 10^2/3^ = 4.64, an applied pressure at the airway of 30 cmH_2_O would result, according to the Mead et al. model, in a local tension approximating a pressure of ~140 cmH_2_O applied to a fully homogeneous and open lung. When we estimated lung inhomogeneity with a CT scan, we found that the multiplication factor between units with different volumes is ~2, but more than enough to locally expand some units to their own TLC [66]. More than 40% of the lung volume in severe ARDS may be subject to this stress-raising phenomenon, emphasizing the importance of designing maneuvers able to decrease lung inhomogeneity.

### Ventilator-related causes of VILI: the mechanical power

**Fig. 3:**
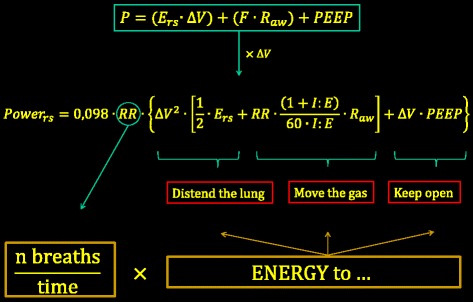
*Upper box*: simplified equation of motion, showing that, at any given moment, the pressure in the respiratory system (*P*) above the relaxed volume equals the sum of the elastic pressure (elastance of the respiratory system *E*_*rs*_ times change in lung volume), plus the pressure needed to move the gases (flow *F* times airway resistance), plus the pressure (if any) to keep the lung pressure above the atmospheric pressure at end expiration (PEEP). If each of these three components is multiplied by the tidal change in lung volume ∆*V*, the energy per breath is obtained. If multiplied by the respiratory rate, the corresponding power equation is obtained. 0.098 is the conversion factor from liters/cmH_2_O to Joules (J). *I:E* inspiratory–expiratory ratio, *PEEP* positive end-expiratory pressure, *Power*_*rs*_ mechanical power to the respiratory system, *RR* respiratory rate, ∆V change of volume *R*_*aw*_ airways resistances

All of these mechanical factors discussed separately (volume, pressure, rate, and flow) can be considered parts of a single physical entity: the mechanical power. The equation describing power (Fig. 3) may be easily derived by multiplying the classical equation of motion by the tidal volume and respiratory rate [67]. Indeed, the energy cost per cycle is computed as the product of pressure times the change of volume, which, when multiplied by the respiratory rate, gives the power value (energy/unit of time). Total pressure is spent in performing elastic work (elastance times tidal volume), in moving gas (flow times resistance), and in maintaining end-expiratory lung volume (by PEEP). If each of these elements is multiplied by the tidal volume, the energy per breath is obtained, and by multiplying this by the respiratory rate we obtain the mechanical power. This equation is presented in this extended form, instead of other possible simplified versions [67], to illustrate item by item the determinants of power. A comparison of exponents indicates that tidal volume (and its associated driving pressure) and inspiratory flow are quantitatively potent determinants (${Power}_{rs}= k*\Delta {V}^2$ and ${Power}_{rs}= k*{flow}^2$), followed by the respiratory rate (${Power}_{rs}= k*{RR}^{1.4}$), and then by PEEP, elastance, and resistance (all three linearly correlated with the mechanical power). Clearly, reduction of ventilatory demand to reduce tidal volume, flow, and/or respiratory rate should be prioritized if applying damaging power is to be avoided.

**Fig. 4:**
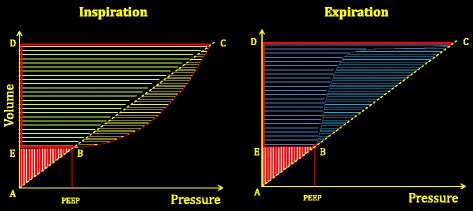
*Left*: baseline energy (*red hatched triangle ABE*), on which the inspiratory energy associated with the tidal volume (area *BCDE*) is added. *Yellow hatched area* to the right of line *BC* represents the inspiratory dissipated energy needed to move the gas, to overcome surface tension forces, to make the extracellular sheets slide across one another (tissue resistances), and possibly to reinflate collapsed pulmonary units. *Light green hatched area* on the left of line *BC* defines the elastic energy (trapezoid *EBCD*) cyclically added to the respiratory system during inspiration. Total area included in the triangle *ACD* is the total energy level present in the respiratory system at end inspiration. *Right*: energy changes during expiration. Of the total energy accumulated at end inspiration (triangle *ACD*), the area of the trapezoid *EBCD* is the energy released during expiration. The fraction of energy included in the hysteresis area (*light blue hatched area*) is dissipated into the respiratory system, while the remaining area (*dark blue hatched area*) is energy dissipated into the atmosphere through the connecting circuit. Note that whatever maneuver (as controlled expiration) reduces the hysteresis area will reduce the energy dissipated into the respiratory system (potentially dangerous?). *PEEP* positive end-expiratory pressure (Color figure online)

Although the concept of mechanical power may appeal as a unifying variable with which to track VILI risk (both during controlled and spontaneously assisted breathing), several challenges must be met before it can be implemented in practice: first, power must be normalized either for a standard lung volume or for the amount of aerated lung tissue [68, 69]; and second, the relationship between the power delivered to the whole respiratory system and that actually delivered to the lung (using the transpulmonary pressure) must be differentiated. In particular, the impact of inspiratory flow and tissue resistance should be better defined. From a practical perspective, even if appropriately adjusted for resistance, flow, and chest wall elastance, any estimate of lung-delivered power made using airway pressure alone during spontaneous efforts would reflect only the machine’s contribution to the total energy imparted during inflation [33]. In addition, the distribution of mechanical power throughout the lung parenchyma must be determined. We do not know whether it follows the same maldistribution of stress and strain dictated by lung inhomogeneity [66]. Finally, mechanical power as defined here relates to the inspiratory phase; it is very possible that the expiratory phase may also play a role. Indeed, all of the energy accumulated at end inspiration must have dissipated both into the lung structures and the atmosphere when exhalation is complete. It is interesting and potentially important to know whether controlling expiratory flow (which decreases the fraction of energy expended into the lung) thereby helps to reduce VILI. Actually, such a phenomenon has been reported in two studies not normally considered in the VILI literature [70, 71]. Fig. 4 summarizes all of these concepts, and also suggests a slightly different nomenclature which we believe to be less confusing than that currently employed.

## Conclusion

To minimize adverse interactions between lung pathology and ventilatory settings that promote VILI requires two distinct strategies: on one side, decreasing the inspiratory (and possibly the expiratory) mechanical power and damaging strain should decrease VILI; and on the other, steps to increase lung homogeneity should decrease the likelihood of injury. The best available maneuver to encourage mechanical homogeneity, supported by solid pathophysiological background [72] and proven clinical results, is prone positioning for those patients in whom inhomogeneity is prevalent (moderate-severe and severe ARDS) [73].

In conclusion, we believe that a possible pathway toward ‘improved’ mechanical ventilation for a future patient would consist of the following steps:

- Define excessive strain and mechanical power, normalized for lung volume.
- Measure/estimate lung inhomogeneity to assess the prevalence of stress raisers and the distribution of mechanical power/stress–strain.
- Determine whether a given ventilatory set applied to the lung parenchyma of which the mechanical characteristics are known is associated with risk of VILI and how much.
- If a mechanical ventilation set cannot be found to avoid an excessive risk of VILI, alternative methods (as the artificial lung) should be considered.

## Abbreviations

∆*V*: change of volume
ARDS: Acute respiratory distress syndrome
ARMA: Low tidal volume trial of the ARDS Network
*bpm*: breaths per minute
*CO*_2_: Carbon dioxide
ECMO: Extracorporeal membrane oxygenation
*E*_L_: Lung elastance
*E*_Lspec_: Specific lung elastance
*E*_tot_: Total elastance of the respiratory system
*E*_w_: Chest wall elastance
*FRC*: Functional residual capacity
*PaCO*_2_: Arterial partial pressure of carbon dioxide
*P*_aw_: Airway pressure
*PBW*: Predicted body weight
*PEEP*: Positive end-expiratory pressure
*P*_L_: Transpulmonary pressure
*P*_musc_: Pressure generated by the respiratory muscles
*Power*_rs_: Mechanical power to the respiratory system
*P*_pl_: Pleural pressure
*RR*: Respiratory rate
VILI: Ventilator-induced lung injury
